# Association of Hemoglobin A1c Levels With All-Cause Mortality in Patients With Coronary Artery Disease: The Essen Coronary Artery Disease Registry

**DOI:** 10.1016/j.jacadv.2025.101624

**Published:** 2025-02-21

**Authors:** Iryna Dykun, Natalie Kappo, Jürgen Kampf, Olga Babinets, R. Alexander Jánosi, Matthias Totzeck, Tienush Rassaf, Amir A. Mahabadi

**Affiliations:** Department of Cardiology and Vascular Medicine, University of Duisburg-Essen, Essen, Germany

**Keywords:** coronary artery disease, diabetes mellitus, HbA1c, outcome

## Abstract

**Background:**

Hemoglobin A1c (HbA1c) as a marker of long-term glycemic control is used for the diagnosis and assessment of treatment of diabetes mellitus.

**Objectives:**

The authors aimed to evaluate whether HbA1c levels would independently associate with long-term survival independent of the presence of other cardiovascular risk factors.

**Methods:**

The present retrospective analysis is based on the longitudinal the Essen Coronary Artery Disease registry of consecutive patients undergoing coronary revascularization therapy between 2004 and 2019. Cox regression analysis was used to determine the association of HbA1c with all-cause mortality. To assess for nonlinearity between HbA1c as a continuous variable and all-cause mortality, we fitted restricted cubic spline models. Kaplan-Meier analysis was used to depict the survival probability.

**Results:**

Overall, 4,700 patients (mean age 66.1 ± 11.4 years, 77.1% male) were included. Mean HbA1c was 6.3% ± 1.2%. During a median follow-up of 2.8 (IQR: 0.6-6.2) years, 558 patients (8.4%) died. In multivariable analysis, higher HbA1c levels, adjusted for other cardiovascular risk factors, were independently associated with all-cause mortality (HR: 1.16 [95% CI: 1.07-1.26] per 1 SD change in HbA1c, *P* < 0.001). Using HbA1c >5.3% to 5.6% as reference, we observed a U-shaped event rate for different HbA1c groups (≤5.3%: HR: 1.52 [95% CI: 1.01-2.28], *P* = 0.0044; >5.6%-5.9%: HR: 0.96 [95% CI: 0.65-1.41], *P* = 0.80; >5.9%-6.6%: HR: 1.26 [95% CI: 0.90-1.78], *P* = 0.20; >6.6-7.8: HR: 1.38 [95% CI: 0.93-2.04], *P* = 0.10; ≥7.8%: HR: 2.15 [95% CI: 1.43-3.19], *P* < 0.001).

**Conclusions:**

In the present large registry of patients with established coronary artery disease (CAD) undergoing coronary revascularization therapy, HbA1c levels display a U-shaped association with all-cause mortality. In addition to patients with diabetes, also patients with CAD with very low HbA1c face increased mortality risk. Clinical trials, randomizing to different HbA1c targets are needed to define optimal HbA1c targets for patients with established CAD.

Hemoglobin A1c (HbA1c) as a marker of long-term glycemic control is used in clinical routine for the diagnosis and assessment of treatment of diabetes mellitus.^1^^,^^2^ Increased HbA1c levels are associated with a higher risk of incident cardiovascular disease in patients both with and without prevalent diabetes.^2^, ^3^, ^4^, ^5^, ^6^, ^7^ However, patients with elevated HbA1c frequently present with other atherogenic risk factors including obesity, dyslipidemia, hypertension, and smoking, which similarly increase cardiovascular disease risk.^8^, ^9^, ^10^ Studies on serial intravascular ultrasound showed that intensified lipid lowering therapy can alter the disease progression in diabetic cohorts and even lead to plaque regression.^11^^,^^12^ A recent post hoc pooled analysis of data from 7 prospective randomized controlled trials involving serial coronary intravascular ultrasound showed that greater HbA1c levels significantly associated with coronary atheroma progression rates independent of achieved cardiovascular risk factor control, suggesting a direct specific effect of glycemic control upon coronary atheroma progression.^13^ Whether this link between HbA1c levels and atheroma progression also transfers into survival of patients with established coronary artery disease (CAD), independent of control of other cardiovascular risk factors, has not been evaluated.

Therefore, the present longitudinal registry of patients receiving interventional coronary revascularization aimed to evaluate whether HbA1c levels would associate with long-term survival independent of the presence of other cardiovascular risk factors.

## Methods

### Study population

The present analysis is based on a retrospective registry of consecutive patients undergoing conventional coronary angiography at the West German Heart and Vascular Center, ECAD (Essen Coronary Artery Disease registry; NCT04196712).^14^ For the present analysis, only patients receiving interventional coronary revascularization therapy between January 1, 2004, and July 23, 2019 were included. Of the 40,461 patients who underwent coronary angiography in the timeframe, there were 12,904 subjects with available HbA1c data. Excluding patients who did not receive percutaneous coronary intervention (n = 8,088), a cohort of 4,700 unique patients was identified (Supplemental Figure 1). This analysis was conducted in accordance with the Strengthening the Reporting of Observational Studies in Epidemiology guidelines.^15^ The Strengthening the Reporting of Observational Studies in Epidemiology checklist (Supplemental Table 1) was used to ensure comprehensive reporting of the study design, implementation, and findings.

### Clinical characteristics and covariate assessment

Information on traditional cardiovascular risk factors from the same hospital stay was drawn from the hospital information system and merged into the database. Laboratory variables were measured using standardized enzymatic methods (low- and high-density lipoprotein cholesterol [HDL-C], lipoprotein(a), C-reactive protein [CRP], and creatinine) and automatically imported. HbA1c levels were quantified using the automated glycohemoglobin analyzer HPLC G8 ZL02/40-01 (Tosoh Medics) at hospital admission. Self-reported information on current smoking status and family history of premature CAD was classified as present, absent, or unknown. Medication information was drawn from discharge letters. Systolic blood pressure on the admission was used for the analysis. Antihypertensive therapy was defined as medication with angiotensin-converting enzyme inhibitors, angiotensin II receptor blockers, calcium-channel blockers, diuretics, beta-blockers, and/or alpha blockers. Hypertension was defined as systolic blood pressure of >140 mm Hg, diastolic blood pressure of >90 mm Hg, or receiving antihypertensive medication. Primary discharge diagnoses were obtained from the hospital information system according to the International Classification of Diseases-10th Revision. Primary diagnosis of CAD was defined as International Classification of Diseases (ICD) codes from I20.0 to I25.9. Acute coronary syndrome was defined as ICD codes I20.0 to 24.9, whereas ICD codes I25.0 to 25.9 were defined as chronic coronary syndrome.

### Endpoint definition

All-cause mortality was defined as the primary endpoint variable. Information on survival status was assessed from all available hospital records (including partner health care facilities) as well as insurance information. Any ambulatory or inpatient presentation to the West German Heart and Vascular Center, the University Hospital Essen or any partner health care facility after the coronary exam was used for confirmation of survival status. Patients without confirmed death but no recurrent presentation to the health care provider were considered lost to follow-up and excluded from the present analysis.

### Statistical analysis

Continuous variables are reported as mean ± SD when normally distributed and as median (IQR) when non-normally distributed. Categorical variables are reported as frequencies and percentages. Two-sided *t*-tests were used for normally distributed continuous variables, and chi-square tests were used for categorical variables. The incidence of death from any cause during follow-up was recorded. To assess for nonlinearity between HbA1c as a continuous variable and all-cause mortality, we fitted restricted cubic spline models with 3 knots at HbA1c values of 6%, 7%, and 8% after adjusting for sex and age. Kaplan-Meier analysis was used to depict the survival probability, stratified across percentiles of patients' HbA1c level (<10th, 10th-<25th, 25th-<50th, 50th-<75th, 75th-<90th, ≥90th percentile). Differences between the groups were evaluated using the log rank test. The association of HbA1c and all-cause mortality was examined using Cox proportional hazards models in the overall cohort. This time HbA1c entered the model directly, neither through groups nor through splines. HR with 95% CIs was reported per 1 SD increase in HbA1c. The proportional hazard assumption was tested by the Schoenfeld residual test. Sensitivity analyses were performed stratifying across percentiles of patients' HbA1c level as well as clinically established HbA1c thresholds. Furthermore, we also performed a sensitivity analysis including all patients of the ECAD registry that received coronary angiography, also including patients without interventional revascularization therapy. A multivariable model included adjustments for age, sex, low-density lipoprotein cholesterol (LDL-C), HDL-C, presence of hypertension, family history of premature CAD, and present smoking status. Data on all variables included in the multivariable model were available for all patients. A Forest plot illustrates the association of HbA1c with all-cause mortality in different subgroups depending on the presence/absence of other risk factors. These include groups of patients <65 vs ≥65 years of age, LDL-C ≥100 or <100 mg/dL, HDL-C < or ≥ than its median level, CRP <2.0 vs ≥2.0 mg/dl, systolic blood pressure >130 or ≤130 mm Hg, smokers or nonsmokers, and presence or absence of Heart failure or acute coronary syndrome at baseline. Cox regression modeling was performed with the same adjustments as outlined above in the original multivariable model. All tests were 2-tailed with a 0.05 significance level. Analyses were performed using SAS version 9.4 (SAS Institute Inc) and R 4.2.0 (The R Foundation for Statistical Computing). The Forest plot was created using Excel (version 16.43, Microsoft).

## Results

Overall, 4,700 patients were included in the present analysis. During a median follow-up of 2.8 (IQR: 0.6-6.2) years, 558 patients (11.9%) died. Mean overall age was 66.1 ± 11.4 years, 22.9% were women and mean HbA1c was 6.3% ± 1.2%. Table 1 describes baseline clinical characteristics of the study population, stratified by survival status. Survivors were younger, had higher HDL-C, lower HbA1c, CRP, and Creatinine levels, had higher frequency of family history of premature CAD, and were less frequent current smokers than nonsurvivors. Presentation with chronic coronary syndrome was more frequent among survivors.

**Table 1:** Baseline Characteristics

	Overall (N = 4,700)	Nonsurvivor (n = 558)	Survivor (n = 4,142)	*P* Value (Between Groups)
Demographics				
Age, y	66.1 ± 11.4	70.1 ± 10.8	65.5 ± 11.3	<0.001
Male	3,625 (77.1)	442 (79.2)	3,183 (76.9)	0.20
Cardiovascular risk factors				
Family CAD history	914 (19.5)	98 (17.6)	816 (19.7)	<0.001
Current smoker	615 (13.1)	81 (14.5)	534 (12.9)	<0.001
Systolic blood pressure	137.2 ± 21.9	136.3 ± 22.9	137.3 ± 21.8	0.30
Hypertension	3,356 (71.4)	404 (72.4)	2,952 (71.3)	0.60
Laboratory measurements				
LDL, mg/dL	104.9 ± 38.8	102.1 ± 42.4	105.3 ± 38.3	0.09
HDL, mg/dL	46.6 ± 13.9	43.7 ± 14.1	47.0 ± 13.8	<0.001
LP(a), mg/dL	16 (7-58)	20 (7-57)	16 (7-58)	0.40^a^
HbA1c, %	6.28 ± 1.19	6.41 ± 1.25	6.26 ± 1.18	0.009
CRP, mg/dL	0.6 (0.1-1.3)	0.9 (0.3-2.2)	0.6 (0.1-1.2)	<0.001^a^
Creatinine, mg/dL	1.31 ± 0.75	1.58 ± 1.06	1.28 ± 0.69	<0.001
Hb, g/dL	13.7 ± 1.7	13.8 ± 1.7	13.1 ± 1.9	<0.001
Clinical presentation				<0.001
Coronary artery disease	3,452 (73.5)	336 (60.2)	3,116 (75.2)	
Chronic coronary syndrome	2,008 (42.7)	162 (29.0)	1,846 (44.6)	
Unstable angina	741 (15.8)	62 (11.1)	679 (16.4)	
NSTEMI	451 (9.6)	86 (15.4)	365 (8.8)	
STEMI	252 (5.4)	26 (4.7)	226 (5.5)	
Other cardiac diagnosis	476 (10.1)	111 (19.9)	365 (8.8)	
Noncardiac	772 (16.4)	111 (19.9)	661 (16.0)	

Values are mean ± SD, n (%), or median (IQR).

CAD = coronary artery disease; CRP = C-reactive protein; Hb = hemoglobin; HbA1c = hemoglobin A1c; HDL = high-density lipoprotein; LDL = low-density lipoprotein; LP(a) = lipoprotein(a); NSTEMI = non–ST-segment elevation myocardial infarction; STEMI = ST-segment elevation myocardial infarction.

a Nonparametric.

Central Illustration depicts the distribution of HbA1c in the overall cohort as well as the age- and sex-adjusted association between HbA1c on a continuous scale and all-cause mortality using restricted cubic splines. Interestingly, this relationship was not linear, whereas a U-shaped pattern was observed, so that both patients with HbA1c <5.7% and those with HbA1c >6.5% had significantly increased risk for all-cause mortality.

**Central Illustration:**
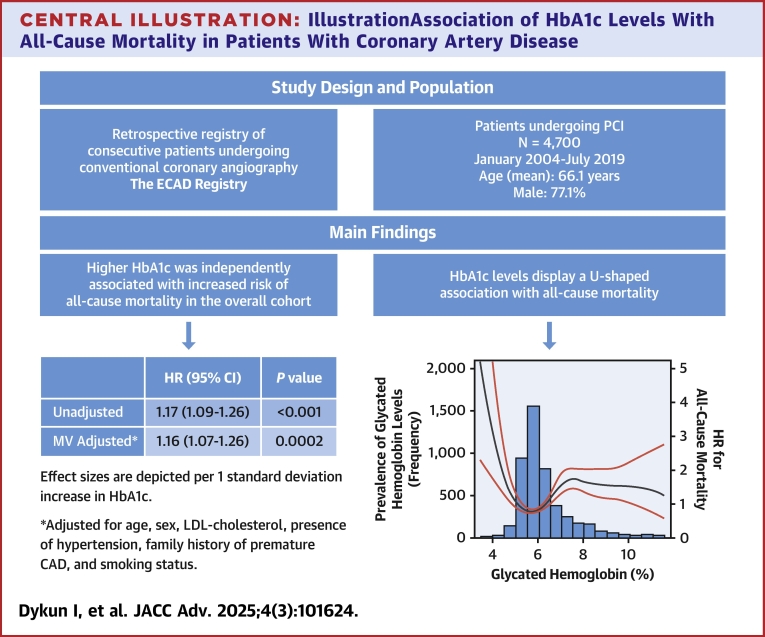
IllustrationAssociation of HbA1c Levels With All-Cause Mortality in Patients With Coronary Artery Disease Distribution of glycated hemoglobin levels and hazard ratios and 95% CIs for its association with all-cause death according to a restricted cubic spline Cox regression model using 3 knots at 6%, 7%, and 8%. Solid black line depicts HR, and solid red lines depict 95% CI. Spline curve is superimposed on histogram showing frequency of glycated hemoglobin (HbA1c) levels in the study cohort (left axis). Effect sizes are depicted per 1 SD increase in HbA1c. ∗Adjusted for age, sex, low-density lipoprotein cholesterol, presence of hypertension, family history of premature coronary artery disease, and smoking status. ECAD = the Essen Coronary Artery Disease; HbA1c = hemoglobin A1c; MV = multivariable; PCI = percutaneous coronary intervention.

**Figure 1:**
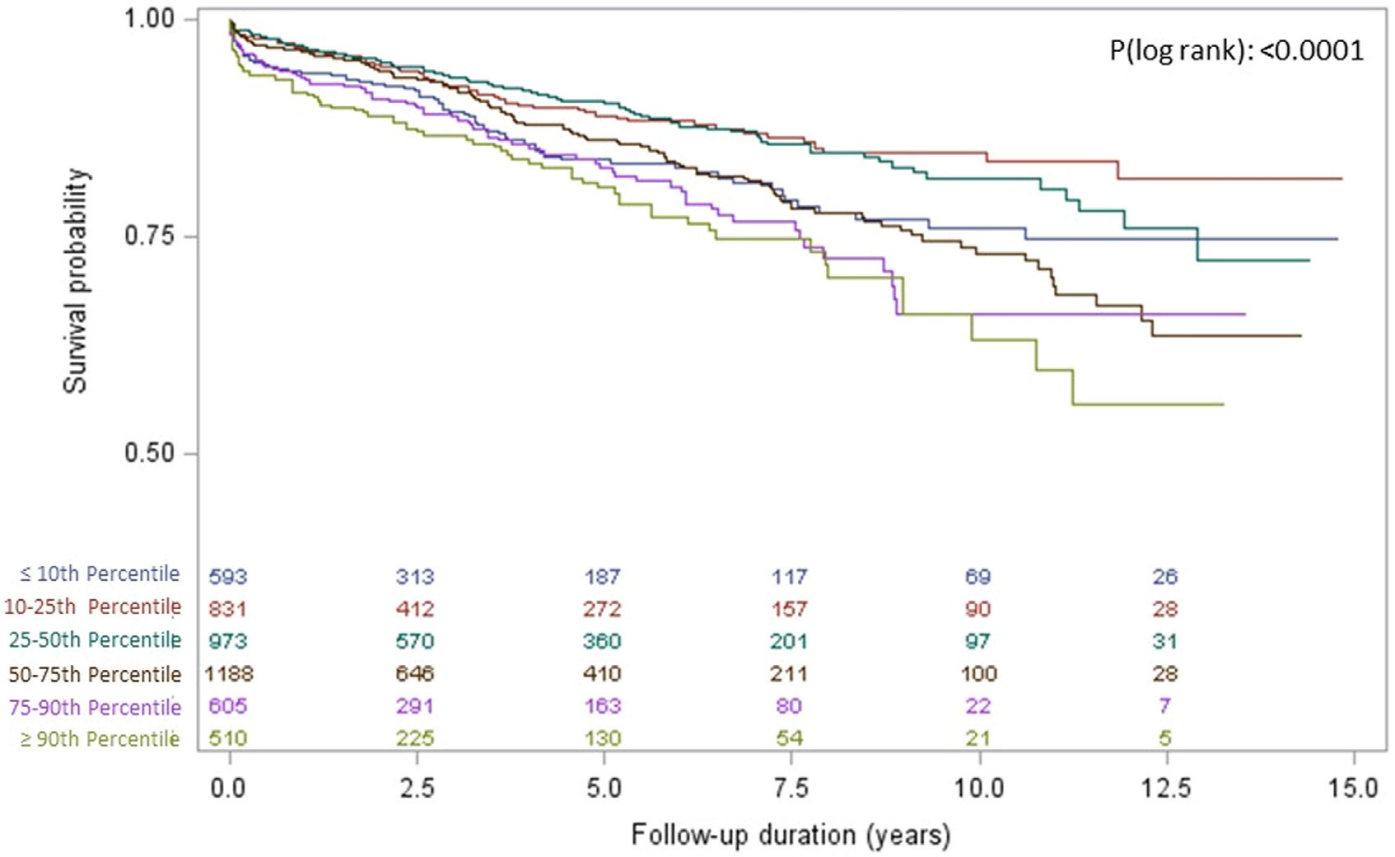
Kaplan-Meier Survival Estimates for the Survival According to Glycated Hemoglobin Levels

Table 2 describes the association of HbA1c as continuous variable with all-cause mortality in unadjusted and multivariable adjusted modeling, this time not using cubic splines. In the multivariable adjusted analysis, higher HbA1c by 1 SD was independently associated with a 16% (7; 26%, *P* < 0.001) increased risk of all-cause mortality in the overall cohort. In the sensitivity analysis also including patients without interventional revascularization therapy, the effect size was even more pronounced (Supplemental Table 2). Figure 1 displays Kaplan-Meier analysis illustrating cumulative incidence of all-cause mortality among patient stratified across percentiles of patients' HbA1c level, confirming the U-shaped association of HbA1c with mortality following coronary intervention. Best long-term survival was observed for the group of patients between the 10th and 25th percentile (HbA1c 5.4%-5.6%), followed by the group of HbA1c between the 25th and 50th percentile (HbA1c 5.7%-5.9%). In contrast, increased mortality was present for patients with both lower (<10th percentile, HbA1c <5.4%) and higher HbA1c levels (≥50th percentile, HbA1c ≥6.0%). Table 3 depicts the Cox regression analysis for the association of HbA1c percentile group with mortality, using the 10th to 25th percentile as reference. We observed that both low (<10th percentile) as well as high HbA1c levels (≥75th percentile) were linked with adverse prognosis in unadjusted an adjusted regression analysis. When using the overall cohort of patients undergoing coronary angiography comparable results were observed (Supplemental Table 3). Similarly, stratifying by HbA1c groups according to established cutoffs, using HbA1c levels of ≥5.7% to 6.0% as reference, both higher and lower HbA1c levels were linked with increased mortality (Supplemental Table 4). Interestingly, for patients with HbA1c levels of 6.0% to 6.5% effect sizes were attenuated when adjusting for age and sex as well as ancillary for other cardiovascular risk factors. In contrast, the higher mortality rate in patients with HbA1c levels of >6.5% persisted upon risk factor control.

**Table 2:** Cox Regression Analysis for the Association of HbA1c as Continuous Variable With All-Cause Mortality

	HR (95% CI)	*P* Value
Unadjusted	1.17 (1.09-1.26)	<0.001
Age- and sex-adjusted	1.17 (1.08-1.26)	<0.001
MV-adjusted^a^	1.16 (1.05-1.28)	0.003

Values are hazard ratios with 95% confidence intervals. Effect sizes are depicted per 1 SD increase in HbA1c.

CAD = coronary artery disease; HbA1c = hemoglobin A1c; HDL-C = high-density lipoprotein cholesterol; LDL-C = low-density lipoprotein cholesterol; MV = multivariable.

a Adjusted for age, sex, LDL-C, HDL-C, presence of hypertension, family history of premature CAD, and smoking status.

**Table 3:** Cox Regression Analysis for the Association of HbA1c Percentile With All-Cause Mortality, Using 10th to 25th Percentile as Reference

	≤10th Percentile HbA1c ≤5.3% (n = 593) 79 Events (13.3%)		10th-25th Percentile HbA1c >5.3%-5.6% (n = 831) 68 Events (8.2%)		25th-50th Percentile HbA1c >5.6%-5.9% (n = 973) 92 Events (9.5%)		50th-75th Percentile HbA1c >5.9%-6.6% (n = 1,188) 151 Events (12.7%)		75th-90th Percentile HbA1c >6.6%-7.8% (n = 605) 86 Events (14.2%)		≥90th Percentile HbA1c ≥7.8% (n = 510) 82 Events (16.1%)	
	HR (95% CI)	*P* Value	HR (95% CI)	*P* Value	HR (95% CI)	*P* Value	HR (95% CI)	*P* Value	HR (95% CI)	*P* Value	HR (95% CI)	*P* Value
Unadjusted	1.57 (1.14-2.18)	0.006	REF	REF	1.05 (0.77-1.44)	0.7	1.51 (1.14-2.02)	0.005	1.90 (1.38-2.62)	<0.001	2.26 (1.64-3.13)	<0.001
Age- and sex-adjusted	1.61 (1.17-2.23)	0.004	REF	REF	1.00 (0.73-1.36)	0.97	1.24 (0.93-1.66)	0.14	1.53 (1.11-2.12)	0.01	2.12 (1.53-2.93)	<0.001
MV-adjusted^a^	1.52 (1.01-2.28)	0.044	REF	REF	0.96 (0.65-1.41)	0.83	1.26 (0.90-1.78)	0.18	1.38 (0.93-2.04)	0.11	2.15 (1.45-3.19)	<0.001

Values are hazard ratios with 95% confidence intervals.

CAD = coronary artery disease; HbA1c = hemoglobin A1c; HDL-C = high-density lipoprotein cholesterol; LDL-C = low-density lipoprotein cholesterol; MV = multivariable.

a Adjusted for age, sex, LDL-C, HDL-C, presence of hypertension, family history of premature CAD, and smoking status.

**Figure 2:**
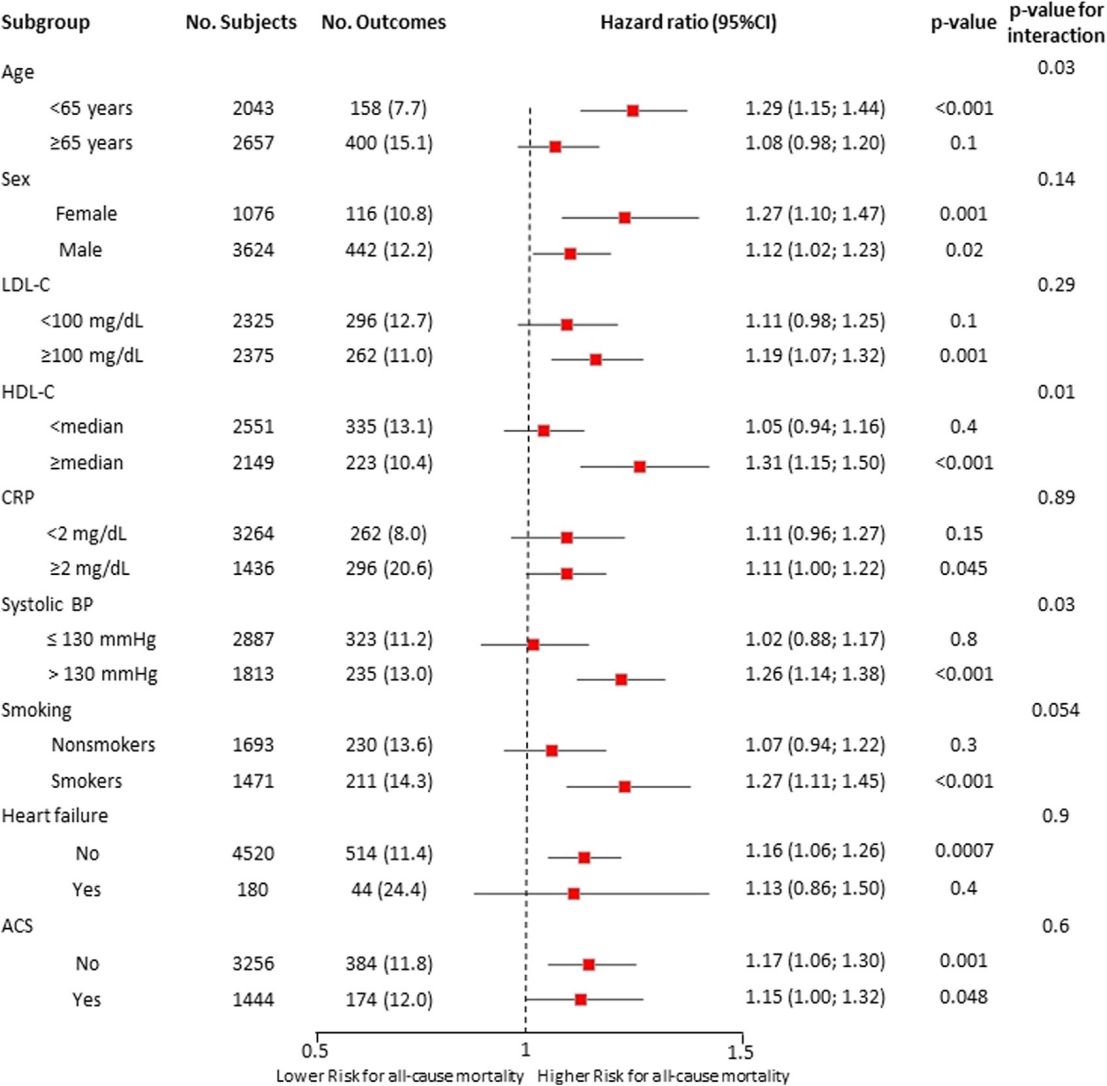
Forest Plot Subgroup analysis of the interaction between traditional risk factors and HbA1c category with the all-cause mortality. BP = blood pressure; CRP = C-reactive protein; HDL-C = high-density lipoprotein cholesterol; LDL-C = low-density lipoprotein cholesterol.

Figure 2 describes a multivariable adjusted model illustrating the relationship between HbA1c levels and all-cause mortality, stratified according to various patient subgroups of interest. Overall, higher levels of HbA1c associated with significantly higher incidence of all-cause mortality, irrespective of sex, LDL-C, CRP levels, smoking status, heart failure, and presence of acute coronary syndrome. Effect sizes were more pronounced in younger patients, and patients with higher high-density lipoprotein (HDL) levels and higher systolic blood pressure.

## Discussion

In the present large longitudinal observational registry of consecutive patients with CAD undergoing interventional coronary revascularization therapy, we demonstrated that: 1) HbA1c levels associate with long-term survival independent of the presence of other cardiovascular risk factors; 2) a stepwise increase in mortality risk with higher HbA1c levels; and 3) that very low HbA1c levels in addition to high HbA1c are linked with adverse prognosis. The observed link between higher HbA1c and mortality risk was more pronounced in patients with HbA1c levels of >6.5%. Together with the previous literature, these data support the notion of glycemic control as an independent marker of risk in patients with established cardiovascular disease despite control of other cardiovascular risk factors.

### HbA1c and mortality risk in patients with CAD

The association of greater HbA1c levels with adverse cardiovascular outcome is well documented in various cohorts.^7^ In the UK General Practice Research Database, poor glycemic control as reflected by high HbA1c levels was associated with increased all-cause mortality in different age groups.^3^ Likewise, investigators of the UK Prospective Diabetes Study found a benefit of improved glycemic control in risk reduction for myocardial infarction and death from any cause.^16^ In contrast, the ARTEMIS Study (Innovation to Reduce Cardiovascular Complications of Diabetes at the Intersection study) showed that cardiac mortality or incidence of MACE in patients with CAD with prediabetes does not differ from those values in patients with normal glycemic status.^17^ A meta-analysis of cardiovascular outcome trials confirmed that intensive glycemic control was associated with a reduction in the risk of MACE during follow-up.^18^ Among patients with and without diabetes mellitus undergoing coronary artery revascularization, increased HbA1c levels were linked with the risk for future myocardial infarction.^19^ This effect may be explained by the effect of HbA1c on coronary atherosclerosis progression, independent from other cardiovascular risk factors, suggesting a pathophysiological link of glycemic control with the progression of disease.^13^ These findings are supported by the present results of a strong association of HbA1c levels with all-cause mortality among patients with established CAD, independent of risk factor control, especially in patients with existing diabetes. However, the present findings question the role of moderately increased HbA1c below the definition of manifest diabetes as marker of increased risk among patients with established CAD. Indeed, we observed comparable mortality rates among patients with optimal HbA1c levels (5.4%-5.6%) as well as patients with mildly elevated HbA1c levels (5.7%-5.9%). In addition, the increased morality rate in the group of patients with HbA1c levels of 6.0% to 6.6% was mainly driven by the presence of concurrent other risk factors, resulting in an attenuation of effect sizes for HbA1c in multivariable models. In the group of patients with HbA1c levels of >6.5%, however, we observed a markedly increase in mortality risk that remained stable upon adjustment for other cardiovascular risk factors. Available data show an inverse association between HbA1c and HDL-C.^20^ In our cohort, we also found lower HDL-levels in nonsurvivors. When evaluating the association of HbA1c with mortality in subgroups of patients with HDL-C of above vs below the median, we detected that the link between glycemic control and mortality risk was more pronounced in patients with high HDL. Further studies are needed to evaluate the precise interaction of HbA1c, HDL, and prognosis in diabetic and nondiabetic cohorts.

### Role of very low HbA1c levels

Besides elevated HbA1c levels, also low HbA1c levels warrant further notification. Malnutrition, inflammation, and functional decline are associated with a decline in HbA1c, especially in elderly patient cohorts.^21^ In the National Health and Nutrition Examination Survey, low HbA1c (defined as HbA1c 4.0%-<5.0%) was associated with a 30% increased risk of all-cause mortality at 5 years as compared to HbA1c levels of 5.0% to <5.7%, while no direct link with cardiovascular endpoints was observed.^22^ In the National Health and Nutrition Examination Survey III, extremely low HbA1c levels of <4.0% showed further increase in mortality risk as compared to HbA1c levels between 5.0% to 5.4%.^23^ Likewise, in an Asian general population cohort, low HbA1c levels associated with both all-cause death and cancer related mortality.^24^ The present results extend the existing literature originating from population based studies to a large cohort of consecutive patients with CAD, confirming the U-shaped association of HbA1c with mortality risk after percutaneous revascularization therapy. Our results underline the need for clinical trials involving patients with established cardiovascular diseases, randomized to different HbA1c targets to define optimal clinical HbA1c targets.

### Study Limitations

Several caveats of the present analysis warrant further consideration. Firstly, our results are based on a single-center experience. While the database includes coronary angiography exams performed by 74 different interventional cardiologists over a time span of 15 years, data originating from other centers and different health care systems need to confirm our results. As by the design of the ECAD registry and the limited information included, we were not able to evaluate, how the dynamic changes of HbA1c levels over time may have influenced the results. In addition, HbA1c levels were available in only parts of the ECAD registry, which may represent a selection bias of included patients. Additionally, regrettably no information on cardiovascular morbidity and mortality is available due to the design of the ECAD registry. Further studies are needed to determine, whether or not the observed effect applies to the complete spectrum of CAD. Lastly, our analysis is based on a predominantly white population; hence, generalization to other ethnic groups remains uncertain.

## Conclusions

In the present large registry of patients with established CAD undergoing coronary revascularization therapy, HbA1c levels display a U-shaped association with all-cause mortality. In addition to patients with diabetes, also patients with CAD with very low HbA1c face increased mortality risk. Clinical trials, randomizing to different HbA1c targets are needed to define optimal HbA1c targets for patients with established CAD.

Perspectives **COMPETENCY IN MEDICAL KNOWLEDGE:** In patients with coronary artery disease undergoing percutaneous coronary intervention, HbA1c is a marker of impaired long-term prognosis, irrespective of the presence of diabetes.

**TRANSLATIONAL OUTLOOK:** Clinical trials, randomizing to different HbA1c targets are needed to define optimal HbA1c targets for patients with established coronary artery disease.

## Funding support and author disclosures

The authors have reported that they have no relationships relevant to the contents of this paper to disclose.
