# Supplementary material for: Association of Hemoglobin A1c Levels With All-Cause Mortality in Patients With Coronary Artery Disease: The Essen Coronary Artery Disease Registry
Source: JACC Adv. 2025 Feb 21;4(3):101624. doi: 10.1016/j.jacadv.2025.101624 (PMC11904530; doi:10.1016/j.jacadv.2025.101624)
Supplement: Supplemental Tables 1, 2, 3, and 4 [file mmc1.pdf]

Online Table 1. STROBE Statement—Checklist of items that should be included in reports of observational studies

	Item No	Recommendation	Page No
**Title and abstract**	1	(*a*) Indicate the study's design with a commonly used term in the title or the abstract	1
		(*b*) Provide in the abstract an informative and balanced summary of what was done and what was found	2
**Introduction**			
Background/rationale	2	Explain the scientific background and rationale for the investigation being reported	4
Objectives	3	State specific objectives, including any prespecified hypotheses	4
**Methods**			
Study design	4	Present key elements of study design early in the paper	5
Setting	5	Describe the setting, locations, and relevant dates, including periods of recruitment, exposure, follow-up, and data collection	5-6
Participants	6	(*a*) Give the eligibility criteria, and the sources and methods of selection of participants. Describe methods of follow-up	5-6
		(*b*) For matched studies, give matching criteria and number of exposed and unexposed	
Variables	7	Clearly define all outcomes, exposures, predictors, potential confounders, and effect modifiers. Give diagnostic criteria, if applicable	6-7
Data sources/ measurement	8*	For each variable of interest, give sources of data and details of methods of assessment (measurement). Describe comparability of assessment methods if there is more than one group	5-6
Bias	9	Describe any efforts to address potential sources of bias	13
Study size	10	Explain how the study size was arrived at	5, Online Figure 1
Quantitative variables	11	Explain how quantitative variables were handled in the analyses. If applicable, describe which groupings were chosen and why	7-8
Statistical methods	12	(*a*) Describe all statistical methods, including those used to control for confounding	7-8
		(*b*) Describe any methods used to examine subgroups and interactions	7-8
		(*c*) Explain how missing data were addressed	7
		(*d*) If applicable, explain how loss to follow-up was addressed	6
		(*e*) Describe any sensitivity analyses	7-8
**Results**			
Participants	13*	(a) Report numbers of individuals at each stage of study—eg numbers potentially eligible, examined for eligibility, confirmed eligible, included in the study, completing follow-up, and analysed	8, Online Figure 1
		(b) Give reasons for non-participation at each stage	NA
		(c) Consider use of a flow diagram	Online Figure 1

Descriptive data	14*	(a) Give characteristics of study participants (eg demographic, clinical, social) and information on exposures and potential confounders	8, Table 1
		(b) Indicate number of participants with missing data for each variable of interest	7
		(c) Summarise follow-up time (eg, average and total amount)	8
Outcome data	15*	Report numbers of outcome events or summary measures over time	8
Main results	16	(*a*) Give unadjusted estimates and, if applicable, confounder-adjusted estimates and their precision (eg, 95% confidence interval). Make clear which confounders were adjusted for and why they were included	9
		(*b*) Report category boundaries when continuous variables were categorized	
		(*c*) If relevant, consider translating estimates of relative risk into absolute risk for a meaningful time period	
Other analyses	17	Report other analyses done—eg analyses of subgroups and interactions, and sensitivity analyses	8-10
**Discussion**			
Key results	18	Summarise key results with reference to study objectives	10
Limitations	19	Discuss limitations of the study, taking into account sources of potential bias or imprecision. Discuss both direction and magnitude of any potential bias	13
Interpretation	20	Give a cautious overall interpretation of results considering objectives, limitations, multiplicity of analyses, results from similar studies, and other relevant evidence	13
Generalisability	21	Discuss the generalisability (external validity) of the study results	10-12
**Other information**			
Funding	22	Give the source of funding and the role of the funders for the present study and, if applicable, for the original study on which the present article is based	1

*Give information separately for exposed and unexposed groups.

Note: An Explanation and Elaboration article discusses each checklist item and gives methodological background and published examples of transparent reporting. The STROBE checklist is best used in conjunction with this article (freely available on the Web sites of PLoS Medicine at http://www.plosmedicine.org/, Annals of Internal Medicine at http://www.annals.org/, and Epidemiology at http://www.epidem.com/). Information on the STROBE Initiative is available at http://www.strobe-statement.org.

Online Table 2. Cox regression analysis for the association of HbA1c as continuous variable with all-cause mortality in patients undergoing coronary angiography n=11437

	Hazard ratio (95% CI)	p-value
Unadjusted	1.21 (1.16; 1.26)	<0.001
Age and sex adjusted	1.20 (1.15; 1.26)	<0.001
MV adjusted*	1.19 (1.13;.1.24)	<0.001

Effect sizes are depicted per 1 standard deviation increase in HbA1c.

*Adjusted for age, sex, LDL-cholesterol, HDL-cholesterol, presence of hypertension, family history of premature CAD, and smoking status.

CAD = coronary artery disease; CI = confidential interval; HbA1c = hemoglobin A1c; HDL-C = high-density lipoprotein cholesterol; LDL-C = low-density lipoprotein cholesterol.

Online Table 3. Cox regression analysis for the association of Hba1c percentile with all-cause mortality, using 10th to 25th percentile as reference in patients undergoing coronary angiography

	**≤ 10th Percentile** HbA1c≤5.3% (n= 1665) Events 229 (13.8%)		**10-25th Percentile** HbA1c>5.3-5.6% (n= 2070) Events 216 (10.4%)		**25-50th Percentile** HbA1c>5.6%-5.9% (n=2373) Events 249 (10.5%)		**50-75th Percentile** HbA1c>5.9-6.6% (n=2875) Events 448 (15.6%)		**75-90th Percentile** HbA1c>6.6-7.8% (n=1406) Events 249 (17.7%)		**≥ 90th Percentile** HbA1c≥7.8% (n=1048) Events 208 (19.9%)	
	Hazard ratio (95% CI)	p-value	Hazard ratio (95% CI)	p-value	Hazard ratio (95% CI)	p-value	Hazard ratio (95% CI)	p-value	Hazard ratio (95% CI)	p-value	Hazard ratio (95% CI)	p-value
Unadjusted	1.30 (1.08; 1.56)	0.006	REF	REF	0.95 (0.79; 1.14)	0.6	1.52 (1.29; 1.79)	<0.001	1.92 (1.60; 2.31)	<0.001	2.24 (1.85; 2.72)	<0.001
Age and sex adjusted	1.39 (1.16; 1.68)	<0.001	REF	REF	0.89 (0.74; 1.07)	0.2	1.30 (1.11; 1.54)	0.002	1.64 (1.36; 1.98)	<0.001	2.14 (1.76; 2.59)	<0.001
MV adjusted*	1.34 (1.10; 1.65)	0.005	REF	REF	0.99 (0.81; 1.20)	0.9	1.34 (1.12; 1.60)	0.001	1.59 (1.29; 1.96)	<0.001	2.08 (1.68; 2.58)	<0.001

*Adjusted for age, sex, LDL-cholesterol, HDL-cholesterol, presence of hypertension, family history of premature CAD, and smoking status.

CAD = coronary artery disease; CI = confidential interval; HbA1c = hemoglobin A1c; HDL-C = high-density lipoprotein cholesterol; LDL-C = low-density lipoprotein cholesterol.

Online Table 4. Cox regression analysis for the association of HbA1c groups (REF: HbA1c ≥5.7-6.0%) with all-cause mortality.

	**HbA1c>5.7% (n= 1424) Events 147 (10.3%)**		**HbA1c ≥5.7-6.0% (n= 973) Events 92 (9.5%)**		**HbA1c ≥6.0%-6.5% (n=972) Events 123 (12.7%)**		**HbA1c ≥ 6.5% (n=1331) Events 196 (14.7%)**	
	Hazard ratio (95% CI)	p-value	Hazard ratio (95% CI)	p-value	Hazard ratio (95% CI)	p-value	Hazard ratio (95% CI)	p-value
Unadjusted	1.18 (0.91; 1.53)	0.2	REF	REF	1.42 (1.08; 1.86)	0.01	1.94 (1.51; 2.49)	<0.001
Age and sex adjusted	1.26 (0.97; 1.64)	0.08	REF	REF	1.25 (0.95; 1.64)	0.1	1.78 (1.39; 2.29)	<0.001
MV adjusted*	1.21 (0.91; 1.60)	0.2	REF	REF	1.15 (0.86; 1.53)	0.3	1.69 (1.30; 2.21)	<0.001

*Adjusted for age, sex, LDL-cholesterol, HDL-cholesterol, presence of hypertension, family history of premature CAD, and smoking status.

CAD = coronary artery disease; CI = confidential interval; HbA1c = hemoglobin A1c; HDL-C = high-density lipoprotein cholesterol; LDL-C = low-density lipoprotein cholesterol.
